# Self-reported extracurricular activity, academic success, and quality of life in UK medical students

**DOI:** 10.5116/ijme.55f8.5f04

**Published:** 2015-09-19

**Authors:** Sophie Lumley, Peter Ward, Lesley Roberts, Jake P Mann

**Affiliations:** 1 Department of medicine, University of Birmingham, UK; 2 Department of medicine, Royal stoke university hospital, UK; 3 Department of medical education, Warwick medical school, university of Warwick, UK; 4 Department of paediatrics, University of Cambridge, UK

**Keywords:** extracurricular activity, academic achievement, quality of life, medical student

## Abstract

**Objectives:**

To explore the relationship between academic performance, extracurricular activity, and quality of life at medical school in the UK to aid our understanding of students’ work-life balance.

**Methods:**

A cross-sectional study, using an electronic questionnaire distributed to UK final year medical students across 20 medical schools (4478 students). Participants reported the hours of self-regulated learning and extracurricular activities undertaken each year at medical school; along with their academic decile (1 = highest, 10 = lowest). Self-reported quality of life (QoL) was assessed using an established screening tool (7 = highest, 1 = lowest).

**Results:**

Seven hundred responses were obtained, across 20 participating medical schools, response rate 16% (700/4478). Factors associated with higher academic achievement were: graduate entry course students (2 deciles higher, p< 0.0001), more hours academic study during term and revision periods (rho=-0.1, p< 0.01), and involvement in teaching or research. Increased hours of study was associated with lower QoL (rho = -0.13, p<0.01).

**Conclusions:**

Study skills may be more important than duration spent studying, for academic achievement and QoL. Graduate-entry students attain higher decile scores despite similar self-reported duration of study.

## Introduction

Medical students’ time is spread across multiple activities: attendance at lectures and placement, exams, extracurricular activities, jobs, family, and social life. Striking a balance between these commitments impacts upon academic performance and quality of life, which also influence each other.

A wealth of factors contribute to academic performance at medical school, including: prior academic performance,^1^^– ^^4^study skills,^5^^,^^6^ aptitude tests (e.g. UKCAT),^7^ attitude, behaviour and motivation,^8^^– ^^12^ time management,^13^^– ^^18^ physical activity,^19^^,^^20^ and coping strategies.^21^^– ^^23^ Whilst academic ability has a significant effect, it is not the sole predictor of achievement at medical school.^16^^,^^24^ Given that A-levels predict future academic performance, but intelligence tests do not, knowledge, motivation, or study skills, might account explain the difference.^25^^– ^^27^ Thus, how students manage their other commitments, study skills, and how they choose to allocate their time may influence their academic outcome. The balance between study, relaxation, and other commitments impacts upon quality of life and preventing burnout.^28^^,^^29^ The high rates of stress, mental health problems, and suicide amongst students and qualified health professionals ^30^^– ^^32^ has been linked to feeling overworked and having long working hours.^33^^,^^34^ For example, misbalancing quantity of sleep with levels of work appears to have a negative impact on quality of life.^35^^– ^^37^ Research into the work-life balance of medical students has implications for students and those involved in their welfare or education. In this study we aimed to answer the following questions:

   •   How do UK medical students divide their time between personal study and extracurricular activities?

   •   What is the relationship between academic success and quality of life?

   •   What other factors influence academic success and quality of life?

## Methods

### Study design

This cross sectional study consisted of a sample of final year medical students across 20 medical schools in the UK, who were asked to complete an online survey between the 15^th^ February and 25^th^ March 2013.

### Participants

Students in the final year of the MBChB (or equivalent) course in the UK and applying to the National Foundation Programme were included in the study. This included graduate-entry students and those who have completed an intercalation course. An ‘intercalation’ is an additional year of study that lies outside the core medical curriculum, often resulting in award of an additional BSc or BMedSc degree. Students currently intercalating were excluded, as they would not be in their final year and not receive a decile score (see below). The study was advertised through emails and on virtual learning environments. Participants were invited to enter a prize draw at the end of the questionnaire. Three randomly selected £50 prizes were awarded.

Ethical approval was given by a key faculty member of each medical school. In addition the project was approved by ethics committees at Birmingham, Hull and York, Imperial College London, Keele, Leeds, Queen Mary (Barts), University College London, University of East Anglia. A participant information sheet was available to all prior to completing the questionnaire. All data provided were anonymous. Institutions were coded before analysis. Prize draw data were collected separately to preserve anonymity, and deleted immediately after the draw.

### Data collection

The questionnaire consisted of 12 closed questions to elicit demographic variables (age and gender), course, academic achievement (decile score), time spent in curricular and extra-curricular activities before final year, leadership, hours of sleep, and QOL scores. Participants listed their demographics and which medical school they attended, then selected which ‘academic decile’ they were ranked in (see below). Participants then gave an average number of hours spent on: self-regulated study and extracurricular activities, for each year of medical school, listing each activity they participated in. Finally, participants responded to 3 “quality of life” statements which were used from a previously published questionnaire that has been validated in an adult population.^38^

 ‘Decile score’ was used as a marker of academic performance. The decile is the rank of performance over the duration of the MBChB/MBBS (or equivalent) course up to the point of application to the Foundation Programme (the first clinical employment after leaving medical school in UK), compared to the graduating cohort. Every final year medical student in the UK is given a decile of 1st-10th. Those in the top 10% of their cohort, according to a range of assessments as defined by their medical school, are in the first decile and those ranked 10-20% are in the second decile, etc. This is calculated by each medical school separately, and does not include intercalated degrees or the final year. Therefore, decile score is medical school-specific i.e. 4th decile at a highly prestigious medical school may equivalent to 1st decile at another medical school. The decile score the main method for comparison of academic performance across UK medical schools; there are no nationalised medical student examinations in the UK.

For this study we defined ‘extracurricular activities’ as: “regular activities that do not directly contribute to course grades and require a degree of skill, service, commitment, or self-discipline”. Participants were asked to estimate the number of hours spent per week in different types of activities for each year of their course, not including intercalated or final years. Students selected from a list of activities (e.g. “Performing arts”, “Sport”, “Music”) and an “Other” table was included for activities that did not fit into the categories. Activities described as “Shopping”, “Socialising” and “Going out” were not included, as they were not felt to demonstrate sufficient commitment, skill or self-discipline. We appreciate that these may be ways of relaxing and balancing academic stress, however they are more difficult to quantify and do not meet our definition of an ‘extracurricular activity’.

A pilot questionnaire was tested on a convenience sample of 14 final year medical students at the University of Birmingham and demonstrated method and content to be feasible and appropriate.

### Sample size and sampling methods

All 33 UK medical schools were invited to participate in the study. 20 medical schools gave approval for. All final year students from included medical schools were invited to participate by email invitation. Two emails were sent to all eligible students during the study period.

### Procedure

Invalid submissions without a decile score, incorrectly completed, or which appeared to be falsified (e.g. total number of hours exceeded number of hours available) were removed from the study. Incomplete responses were included in analysis for the questionnaire sections that had been completed. Invalid answers were removed from the analysis of the respective section.

### Statistical analysis

Categorical data were analysed using χ² and Fishers exact tests. Ordinal data were analysed using Mann-Whitney U-test and univariate analysis was performed using Spearman’s correlation. For all analyses p< 0.05 was considered to indicate statistical significance. Multivariate analysis was performed using linear regression, with decile as the dependent variable and being treated as a continuous variable. Multivariate linear regression was first modelled using all available independent variables. The model was repeated using backwards elimination to exclude redundant independent variables. Statistical and graphical analysis was performed on Graphpad Prism and SPSS v21.0.

## Results

### Demographics

Seven hundred electronic questionnaires were collected, from 4478 students of the participating medical schools; therefore response rate was 16%.

Six hundred and twenty nine out of seven hundred (629/700) were suitable for analysis after exclusions. Four hundred and fifteen (66%) participants were female, and the median age was 23 years (range 22-42 years). Almost all participants were 22 or 23 years old. Responses were obtained from 20 separate medical schools, with a median of 24 responses from each (range 5-80).

Students from all deciles (1-10) responded to the questionnaire. Response frequency appeared to correlate with decile, such that students with higher academic performance responded more often and the fewest responses were from deciles 9 and 10 (see Table 1.)

**Table 1:** Summary survey responses and demographics (N=629)

Variable		
No. female [%]		415 [66]
Median age [range]		23 [22-42]
No. of medical schools		20
Median no. responses per medical school [range]		24 [5-80]
No. graduate-entry students [%]		62 [10]
Median academic decile [range]		4 [1-10]
Hours of self-regulated learning / week [range]		
	Term time	10.6 [0-50]
	Revision period	38.7 [0-120]
Hours of extracurricular activity / week		9.8 [0-69]
Hours of sleep / night [range]		7.2 [5-10]

### Self-regulated learning

Students reported that, across the whole of their medical school study, they engaged in a mean 10.6 hours (quartile-range 5-15hrs) self-regulated learning per week during term time. During dedicated revision periods they undertook a mean of 38.7 hours study per week (quartile-range 22-55hrs). Students who engaged in longer hours of self-regulated learning during the term were also likely to study more hours during revision periods (rho=+0.29, p< 0.0001). A ‘revision period’ is the self-defined time prior to examinations or testing when students would undertake revision, therefore usually associated with an increase in the amount of self-regulated learning.

### Extracurricular activity

Medical students reported a diverse involvement in extracurricular activities, mean 9.8 hours per week (quartile range 1-14 hours), across all of their medical school study. Students spend just under 5-hours per week on sport: the most common extracurricular activity. The type of activity shifted from first year to penultimate year: fewer students were involved in paid employment; more were involved in research, teaching, or participated in committees, although no changes were statistically significant. Overall, 69% of students had held a leadership position in one of their extracurricular activities.

### Quality of life

Students responded to three statements regarding their quality of life (QoL) from 1-7, where “7 = highest quality”. The statements asked participants to what extent they agreed that “life has been close to ideal”, “conditions of life have been excellent”, and “satisfied with my life”. Median score for all was 6 interquartile range 5-7. There was a strong concordance between scores for individual statements (r=0.69-0.77, p<0.0001). Students with better academic performance (lower numerical decile) reported higher quality of life: students in 1st & 2nd deciles were more likely to rate a high QoL (median 7/7) compared to those in 9th & 10th deciles (median 5/7, p<0.0001 by Mann-Whitney U-test).

Spearman’s correlation found hours of sleep to be weakly associated with higher reported QoL (r=0.12, p< 0.01). In contrast, students who engaged in more hours of self-regulated learning during their term reported lower satisfaction with life (r = -0.13, p < 0.01)

### Factors associated with academic decile

Spearman’s correlation analysis found a number of factors to be statistically significantly associated with academic decile. The most significant factor was whether or not the student was on a Graduate Entry Course (GEC), as discussed below (and in Table 4). Better academic performance (lower numerical decile) was seen in students who reported studying more hours during term (r=-0.1, p<0.05) and revision periods (r=-0.1, p< 0.01). There was no relationship between the total hours spent on extracurricular commitments and decile.

Analysis by activity-type demonstrated several statistically-significant correlations. Employment and caring for family was associated with worse academic attainment (r = +0.1, p < 0.05) whereas involvement in research (r=-0.17, p <0.01) or teaching (r=-0.13, p<0.01) were predictors of better performance. Neither gender nor hours of sleep showed a relationship with decile (see Table 2).

**Table 2:** Factors associated with higher academic decile, by Spearman’s correlation

Variable		Spearman’s rho	p
Age		-0.08	<0.05
Hours of self-regulated learning			
	Term time	-0.10	<0.05
	Revision period	-0.10	<0.01
Research activity		-0.17	<0.01
Teaching activity		-0.13	<0.01
Employment		0.10	<0.05
Care for family		0.10	<0.05

### Multivariate analysis

Multivariate linear regression was undertaken to predict decile from all other independent variables (38 total). This model significantly predicted decile, F_(38,445)_=2.706, p< 0.0005, R^2^ = 0.188.

Multivariate linear regression was repeated using backwards elimination to predict decile (see Table 4). All 38 independent variables were initially included, but 8 variables statistically significantly added to the prediction (p< 0.01): age, GEC, involvement in Charity, involvement in Performing arts, Mean hours of extracurricular activity performed per year, conditions of life being Close to Ideal, mean hours in Performing arts, and mean hours in Research. This model significantly predicted decile F_(11, 472)_ = 7.267, p < 0.0005, R^2^ = 0.145 (see Table 3).

**Table 3:** Summary of independent predictors of academic decile on multivariate linear regression with backwards elimination

Variable	Beta	*t*	*p*
Age	-.131	-2.518	.012
GEC	-.131	-2.514	.012
Mean hours of extracurricular activity per week	.133	2.701	.007
Conditions of life being ‘close to Ideal’	-.205	-4.678	.000
Involvement in charitable activity	.757	1.971	.049
Mean hours in performing arts	1.001	2.115	.035
Mean hours in research activity	-.094	-2.059	.040

**Table 4:** Questionnaire responses comparing Graduate Entry Course (GEC) students and non-GEC students

Variable		GEC (n=62)	Non-GEC (n=207)
Mean age [range]		26.5 [24-42]	23 [22-35]
Median decile [range]		2* [1-9]	4* [1-10]
Mean hours of self-regulated learning / week [range]			
	Term	9 [0-32]	10 [0-50]
	Revision	33 [0-100]	35 [0-120]
Mean hours of extracurricular activity / week [range]		10.2 [0-62]	9.8 [0-69]

* p < 0.0001

### Graduate-entry course students

Ten percent (n=62) of the responses were from graduate-entry course (GEC) students. They were on average 3 years older than non-GEC students. GEC students had a median decile of 2 compared to non-GEC students who were in the 4th (median) decile p<0.0001. This was not associated with a difference in the number of hours of self-regulated learning. A similar relationship was demonstrated between decile and age, such that older students were more likely to have higher academic performance (r=-0.08, p< 0.05).

## Discussion

In this study, we describe the association between final year medical students’ self-reported time spent studying, academic performance, and quality of life.

Graduate entry course (GEC) students are, on average, ranked two deciles higher than undergraduate students, in spite of studying for an equivalent number of hours per week. Increasing age and being a graduate-entry student were both independently associated with improved decile on univariable and multivariable analysis. GEC students’ experience and study skills may explain these findings; however they are a select group, having already achieved an undergraduate degree at 1st or 2:1.^39^ Previous research in this field has found variable results comparing undergrate-entry and graduate-entry students.^40^^–^^42^

A number of other factors were found to be associated with higher academic performance. Whilst the magnitude of effect was small they are consistent with those derived by Ferguson et al (2002)^43^ and when combined as a multivariable model we could predict up to 15% of the variability in academic decile. There was a weakly positive association between academic success and number of hours studied. In light of the findings related to GEC students, we could conclude that study skills may be more important than quantity of self-regulated learning, which corroborates with previous reports.^44^^–^^47^

Those engaging in teaching and research as extracurricular activities are also higher academic performers. PAL (Peer-assisted learning) has been highlighted as a way of improving the knowledge of the educators themselves.^48^^,^^49^ Peets et al. focused on retention of the knowledge that the peer-teachers delivered but this study suggests improvement in overall academic outcome. It is possible that students develop transferable skills through teaching and research that can be applied elsewhere. However, this association may occur because the students who are more academically able are those that seek out teaching and or research opportunities.^50^

Evidence suggests that those with poor sleep may have reduced cognitive functioning^51^^,^^52^ and academic performance^53^ but this was not reflected in the findings of this study. However, timing of sleep may be more important than duration^54^ and previous studies into cognitive performance and sleep have looked at short-term effects rather than the long-term academic measurement used in this study.

Time spent on extracurricular activity had minimal association with academic achievement, which is consistent with other reports that maintaining extracurricular activities does not impact upon performance, though stopping such activity may result in worse examination results.^55^ This finding contrasts research conducted at secondary school level, which shows a positive influence.^56^^,^^57^ However the selection involved in gaining a place at medical school limits the range of academic ability, which may explain the change in effect.

There appeared a trend between those achieving better decile and the highest quality of life, consistent with findings elsewhere.^58^ A high score for “The conditions of my life are close to ideal” had the strongest association with improved academic decile on multivariate analysis. However this section of the study is most susceptible to responder bias: students who perform better academically may feel better about their life when reporting it in a research questionnaire. Though stress is associated with poorer exam performance^59^^,^^60^ and there are high rates of mental illness amongst students.^30^^,^^32^ We also acknowledge that students who are unhappy may be missing from this study due to lack of participation.

The findings of this study could have implications for pastoral support services advising students who are struggling, where greater emphasis is placed on study technique than duration spent in self-directed learning. In addition, these results support continued development of graduate-entry medical courses.

### Limitations and further research

The main limitation of this study is the potential for bias. We obtained a greater response rate from students with a higher academic decile therefore our results are skewed towards those with high academic performance and limits the generalisability of the result. The association between response rate and decile may be due to students more willing to disclose their results,^61^ high-performers are more interested in the topic of the questionnaire,^62^ or it may be a reflection engagement with the course and involvement in research.

We obtained responses from 20 of the UK’s 33 medical schools, however our response rate was 16%, which limits the extent that these results can be extrapolated to all UK medical students. The focus of the current study was on self-reported allocation of time, however we didn’t correct for certain co-founders that are known to be associated with academic performance: ethnicity,^63^ socio-economic background^64^, undertaking an intercalacted BSc,^65^^,^^66^ and previous academic results (e.g. A-levels).^67^^,^^68^

All self-reported data has potential for recall bias, particularly when participants were not blinded from the background to the study. We are unable to confirm the accuracy of participant’s recall of their study or extracurricular activity 4 or 5 years previously. We also anticipate that the quality of life assessment will be significantly influenced by the students’ current feelings, rather than recalling them for previous years.

Due to the cross-sectional nature of the study we are able to demonstrate association, not causation. A prospective study would be more effective at showing causation. We have also been unable to account for previous academic performance (e.g. secondary school examination performance).

The comparison of decile ranking is more complex than it first seems. Whilst every student receives a decile of 1st-10th, a 2nd decile at one medical school is not necessarily equivalent, in terms of academic performance, to 2nd decile at another medical school. Also, it is at the discretion of the individual medical school whether to rank the GEC students alongside undergraduates on the same course or only with the GEC cohort.^69^ If present, these effects would be anticipated to ‘dilute’ the magnitude of any differences.

As a result of distributing the questionnaire to final year students we have excluded students who had failed examinations prior to this point. Qualitative assessment through targeted focus groups and interviews could look at work-life balance factors affecting those performing poorly in examinations.

## Conclusions

Graduate-entry course medical students had improved academic performance compared to undergraduate-entry course students, despite reporting equivalent duration on self-directed study. Therefore transferable skills learnt in previous study and in extracurricular research or teaching may aid students’ learning.

### Acknowledgments

Thanks to H. Thyguesen (University of Leeds) for advice on statistical analysis. Thanks to the medical students in all 20 medical schools who helped to distribute the questionnaire

We would like to thank the University of Birmingham student medical society conference committee for their contribution to the prize fund money for this project.

### Conflict of Interest

The authors declare that they have no conflict of interest.
